# Retrospective cohort study of 4,591 dental implants: Analysis of risk indicators for bone loss and prevalence of peri‐implant mucositis and peri‐implantitis

**DOI:** 10.1002/JPER.18-0236

**Published:** 2019-02-06

**Authors:** David French, H. Michelle Grandin, Ronen Ofec

**Affiliations:** ^1^ Faculty of Dentistry Division of Periodontics University of British Columbia Room: JBM 366, 2199 Wesbrook Mall, Vancouver British Columbia Canada; ^2^ Alfred E. Mann Institute University of Southern California Los Angeles CA USA; ^3^ Department of Statistics and Operations research Private Dental practice Tel‐Aviv University Tel‐Aviv Israel

**Keywords:** alveolar bone loss, dental implants, mucositis, peri‐implantitis

## Abstract

**Background:**

Due to the risk of peri‐implantitis, following dental implant placement, this study aimed to evaluate risk indicators associated with marginal bone loss from a retrospective open cohort study of 4,591 dental implants, placed in private practice, with 5‐ to 10‐year follow‐up. Furthermore, the prevalence of mucositis and peri‐implantitis among the study cohort was evaluated, comparing strict versus relaxed criteria for bleeding on probing.

**Methods:**

Periapical radiographs were used to evaluate changes in crestal bone level. Peri‐implant soft tissue was evaluated using an ordinal mucosal index in comparison with the conventional binary threshold for bleeding (i.e., present or not). Periodontal probing depth was not evaluated. Linear mixed models were used to evaluate bone level over time, and other risk indicators, at the patient and implant level.

**Results:**

Risk indicators found to have a significant impact on bone level included: autoimmune disease, heavy smoking, bisphosphonate therapy, implant location, diameter and design, and the presence of a bone defect at site of implantation. The prevalence of mucositis at the implant level was 38.6% versus 14.2% at 6 to 7 years, when using strict versus relaxed criteria, respectively. The prevalence of peri‐implantitis after 6 to 7 years was 4.7% and 3.6% when using strict versus relaxed criteria, respectively.

**Conclusions:**

The results of this study identify several risk factors associated with bone loss. Furthermore, the prevalence of mucositis and peri‐implantitis was shown to be lower at both the implant and the patient when using strict versus relaxed criteria based on the assessment of oral health surrounding dental implants.

## 1 INTRODUCTION

Although dental implants have been reported to have fairly high survival rates of 95.7% at 5 years and 92.8% at 10‐years,^1^ it is also known that progressive marginal bone loss and peri‐implantitis remain a significant potential complication.^1^^‒^ 4 The 2012 European academy of osseointegration (EAO) consensus report estimated the prevalence of peri‐implantitis to be at 10% of implants and 20% of patients, 5 to 10 years after implant placement.^5^ However, there was a wide range in reported prevalence, arising, in part, due to variable implant designs and variability in the thresholds applied for bone loss and soft tissue analysis.

The clinical definition of peri‐implantitis, according to the Sixth European workshop on periodontology, refers to the presence of redness, swelling of mucosa, bleeding and/or suppuration, deepening of pockets adjacent to the implant and loss of supporting bone.^6^ In practice, not every study includes pocket depth measurements, but most studies use marginal bone loss and soft tissue condition as parameters in the classification of peri‐implantitis. The majority of studies determine soft tissue scores using a dichotomous system, e.g.: presence or absence of bleeding‐on‐probing (BOP) or suppuration, though some have used an ordinal BOP scale, thus contributing to the range in reported prevalence.^7^, ^8^

To date, a number of risk indicators have been associated with marginal bone loss. These include patient‐related factors such as smoking, periodontal disease, diabetes, and plaque control/oral hygiene;^9^, ^10^ implant‐related factors such as design of the implant‐abutment complex, and implant shape;^11^^‒^ 13 as well as surgically related factors including the use of bone grafting,^14^ immediate placement,^15^ site preparation and loading,^16^ the degree of separation between implants,^17^, ^18^ the presence of thin mucosal tissue^19^ and soft tissue probing depth.^20^ A further understanding of risk indicators associated with bone loss in private practice would aid in mitigating peri‐implantitis.

The objective of this study was to evaluate risk indicators associated with marginal bone loss as observed in private practice, by evaluating changes in crestal bone level over time, from a retrospective cohort study of an initial 4,591 dental implants of various designs^1^ with a mean of 32.2 months, with some cases up to 5‐ to 10‐years follow‐up. Furthermore, to assess the prevalence of peri‐implantitis, this study aimed to calculate the rate of mucositis and peri‐implantitis using two different thresholds for bleeding on probing from an ordinal scale, while taking care in considering implant design related remodeling and timing of baseline measurements.

## 2 MATERIALS AND METHODS

A description of the study cohort presenting explanatory variables and univariate and multivariate implant survival analysis has been previously published.^21^ Details on recall and follow up are further described in a companion paper.^8^ In brief, this was a retrospective study consisting of 2,060 patients with an initial total of 4,591 implants. All implants were placed between 1999 and 2012, in Calgary, with all surgeries and measurements performed by one periodontist (DF). No intra‐examiner calibration was performed. Implant stability was evaluated at 2 to 3 months post‐insertion, using a 35 Ncm torque test and radiographic bone measurements, which served as baseline for future evaluation of the crestal bone level (CBL). Follow‐up was scheduled at 1‐, 3‐, 5‐ and up to 10 years. Follow‐up was less defined after 5 years as patients generally returned for complications, new surgical site, or were large complex restorations. The study was approved by the Clinical Research Ethics Board at the University of British Columbia (Vancouver # H13‐01664 titled UBC Implants) and was conducted in accordance with the Helsinki Declaration of 1975, as revised in 2000. All patients provided written informed consent to participate in the study.

The majority of the implants were standard design with regular or wide neck, diameters of 4.1 or 4.8 mm and lengths of 8, 10, or 12 mm. All surgeries were performed by open flap using surgical protocols described previously.^21^ All implants, except for immediate socket or bone graft scenarios, were inserted in suitable prosthetic positions with good primary stability and the border between the machined neck and the micro‐rough surface was positioned fully in bone for the circumference of the implant. The impact of guided bone regeneration and immediate socket placement on CBL was evaluated in addition to other risk indicators.

Radiographs were taken and evaluated by the same examiner that placed the implants (DF). For each case the real implant length served as the calibration value to derive the Distance from Implant shoulder to the first Bone to implant contact (DIB).^21^, ^22^ CBL was defined as DIB minus the neck length (NL) of an implant with the following standardization values to account for different implant neck designs designs^2^: 2.8 mm for standard tissue level, 1.8 mm for standard plus tissue level and tapered effect, and 0 mm for bone level implants (see supplementary Figure 1 in online *Journal of Periodontology*). The CBL was a single score recorded as the greatest value from either the mesial or distal measurement as determined by examiner at each recall. Marginal bone loss (MBL) was defined as the change in CBL between subsequent time points during follow‐up, using stage 2, i.e.: 3 months post‐installation, as the baseline.

The risk indicators evaluated in relationship to MBL included 1) pre‐existing disease including autoimmune, diabetes types 1 and 2 and history of periodontitis, 2) heavy smoking (>15 cigarettes/day) and 3) bisphosphonate use, 4) implant location, 5) diameter, 6) implant design, 7) immediate loading, 8) bone defect (=GBR), and 9) insertion torque.

Peri‐implant soft tissue was evaluated by probing with a light vertical probe force of 17 g using a calibrated force automated probe^3^ or manual probe calibrated to about 17 g at six locations around the implant (mb, b, db, ml, l, dl) m = mesial, b = buccal, d = distal, l = lingual.^8^, ^23^ The soft tissue condition based on probing was determined using the Implant Mucosal Index (IMI) which is a modification of the SBi^7^ whereby 0 = no bleeding, 1 = minimal single‐point bleeding, 2 = moderate multi‐point bleeding, 3 = profuse multi‐point bleeding, and 4 = suppuration.^8^ Mucositis was determined using either the “strict” criteria, IMI ≥1, as an indication for mucositis or the “relaxed” criteria, IMI ≥2, as an indication of mucositis. We defined peri‐implantitis as the combination of mucositis and MBL ≥1.0 mm, at least 1 year after installation.

### 2.1 Statistical analysis

CBL and MBL are scale variables and have been summarized by calculating the mean and median as central tendency statistics and the standard deviation, range and percentiles, as dispersion statistics. Linear mixed models were used in order to evaluate CBL as the main outcome variable as a function of time as well as the other explanatory variables. The results from the final model (Table 1) allowed us to test three null hypotheses (H_0_): 1) Mean crestal bone level is equal at start, 2) Mean crestal bone level over time (profile) is equal, and 3) No interaction between bone level and time exist. See supplementary text in online *Journal of Periodontology* for further details and for patient level analysis.

**Table 1:** Explanatory variables having a statistically significant effect on mean crestal bone level over time

		*P* value		
Level	Variable	Difference at start(*)	Main effect(†)	Significant interaction with time(‡)
Patient	Autoimmune	0.21	0.04	No
	Smoking	0.92	<0.01	Yes
	Bisphosphonate	0.04	0.045	No
Implant	Location	<0.01(§)	<0.01	Yes
	Diameter	<0.01	<0.01	Yes
	Implant design	<0.01(‖)	<0.01	No
	Immediate loading	<0.01	<0.01	No
	Bone defect	<0.01	<0.01	yes

NB: The numbers in the column “Difference at start” are the P values testing the null hypothesis (H0) which states that bone level is equal at the start (actually at stage 2 = 3 months) for different categories within a variable. The *P* values in the “Main effect” column indicate whether bone levels over time are different between categories of a variable (i.e.: differences in mean bone loss over the time points recorded). The column “Significant interaction with time” refers to a comparison of slopes between categories of a given variable.

^*^H_0_: Mean crestal bone level is equal at start.

^†^H_0_: Mean crestal bone level over time (profile) is equal.

^‡^H_0_: There is no interaction between bone loss and time (the rate of bone loss is constant over time).

^§^The difference between posterior maxilla and posterior mandible is non‐significant.

^‖^The difference between standard and standard plus, and between bone level and tapered is non‐significant.

In order to calculate the prevalence of mucositis and peri‐implantitis at the implant level, mucositis (as either IMI ≥1 or IMI ≥2) and MBL ≥1 mm were cross‐tabulated at 2 to 3 years, 4 to 5 years, 6 to 7 years, and 8 to 10 years.

The statistical analysis was performed with SPSS^4^ and with R software.^5^ The significance level was set to 0.01.

## 3 RESULTS

The study cohort of 2,060 patients and 4,591 implants was followed for up to 133 months, with a mean of 32.2 ± 26.8 months. The number of implants for each time period was; n = 2,372 at 2 to 3 years, n = 1,178 at 4 to 5 years, and n = 560 at 6 to 10 years. There were 32 implant failures resulting in cumulative survival rates of 99.3%, 99.0%, and 98.4% at 3, 5, and 7 years, respectively, as previously reported.^21^ Of 32 failures recorded, 22 occurred before loading. Of the 10 failures that occurred after loading, four implants were related to peri‐implantitis and six implants failed in relationship to biomechanical load. For the current analysis the 32 failing implants were excluded; therefore, the analyzed cohort included 4,559 implants. Bone measurements were performed at 3 months then at years 1, 2 to 3, 4 to 5, 6 to 7, and up to 8 to 10 years after installation. Over the study period, the mean CBL increased from 0.06 ± 0.22 mm at stage 2 to 0.44 ± 0.81 mm at 8 to 10 years. Throughout the study period, the median CBL was 0 mm. At 8 to 10 years, 15% of implants exhibited a CBL >1.02 mm and 5% exhibited a CBL >2.28 mm.^8^ The number of sites evaluated for each recall period (n, mean CBL ± SD) were: at stage 2, n = 4,524, CBL = 0.06±0.22; at 1‐year, n = 3,532, CBL = 0.13±0.31; at 2 to 3 years, n = 2,372, CBL = 0.16±0.37; at 4 to 5 years, n = 1,178, CBL = 0.21±0.45; at 6 to 7 years, n = 389, CBL = 0.34±0.62, and at 8 to 10 years, n = 171, CBL = 0.44±0.81.

### 3.1 Risk indicators for bone loss (MBL)

**Figure 1:**
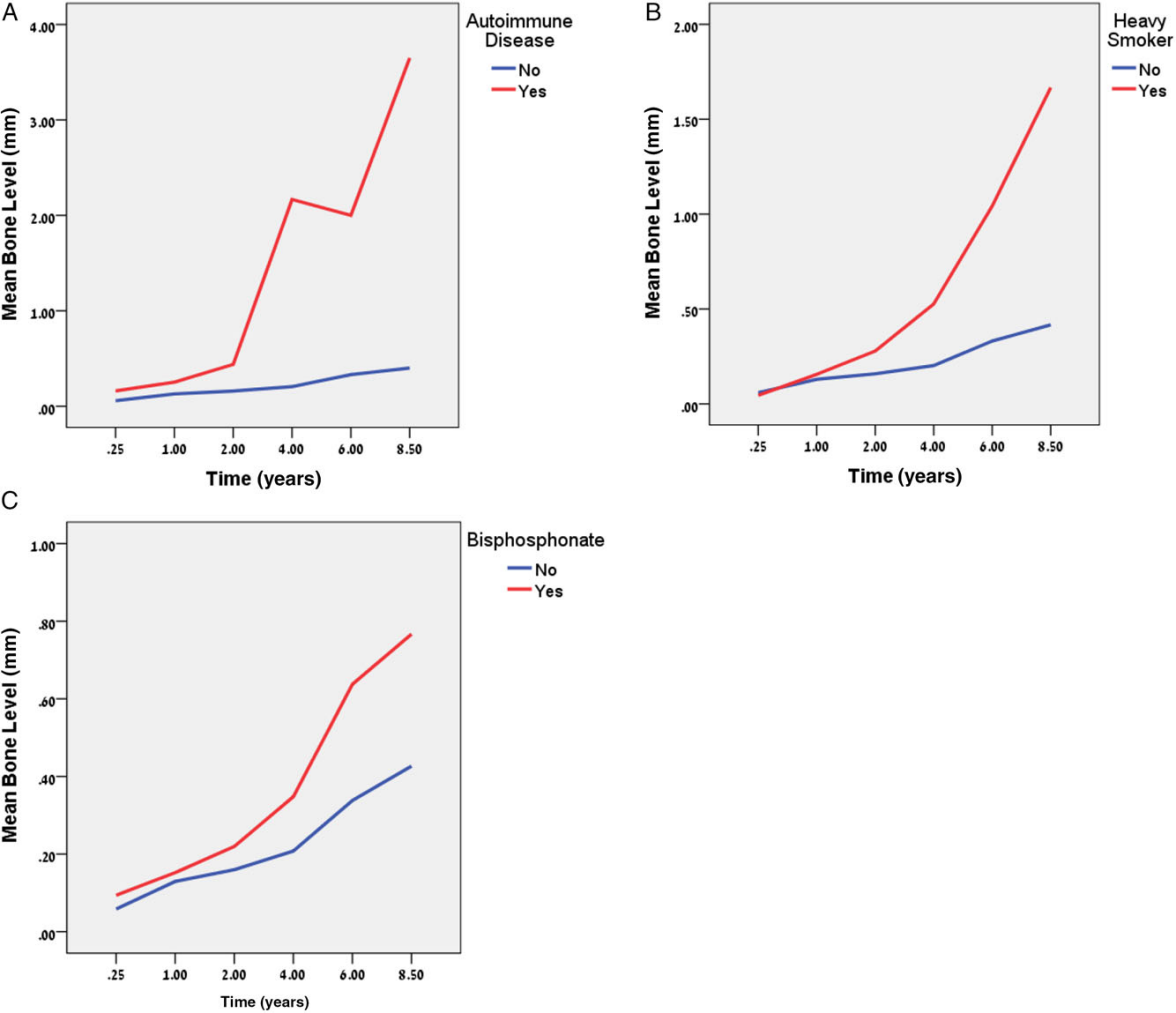
Bone level profiles^*^ for patient‐related factors **A**) autoimmune disease, **B**) smoking status, and **C**) bisphosphonate use. * y‐axis is not the same for different panels, therefore comparison between variables should be avoided

All potential factors and related correlations were evaluated. Table 1 shows only variables that related significantly to changes in CBL over time in a multivariate model. Figures 1 through 3 illustrate the results of Table 1. The “start” refers to baseline at 3 months).

**Figure 2:**
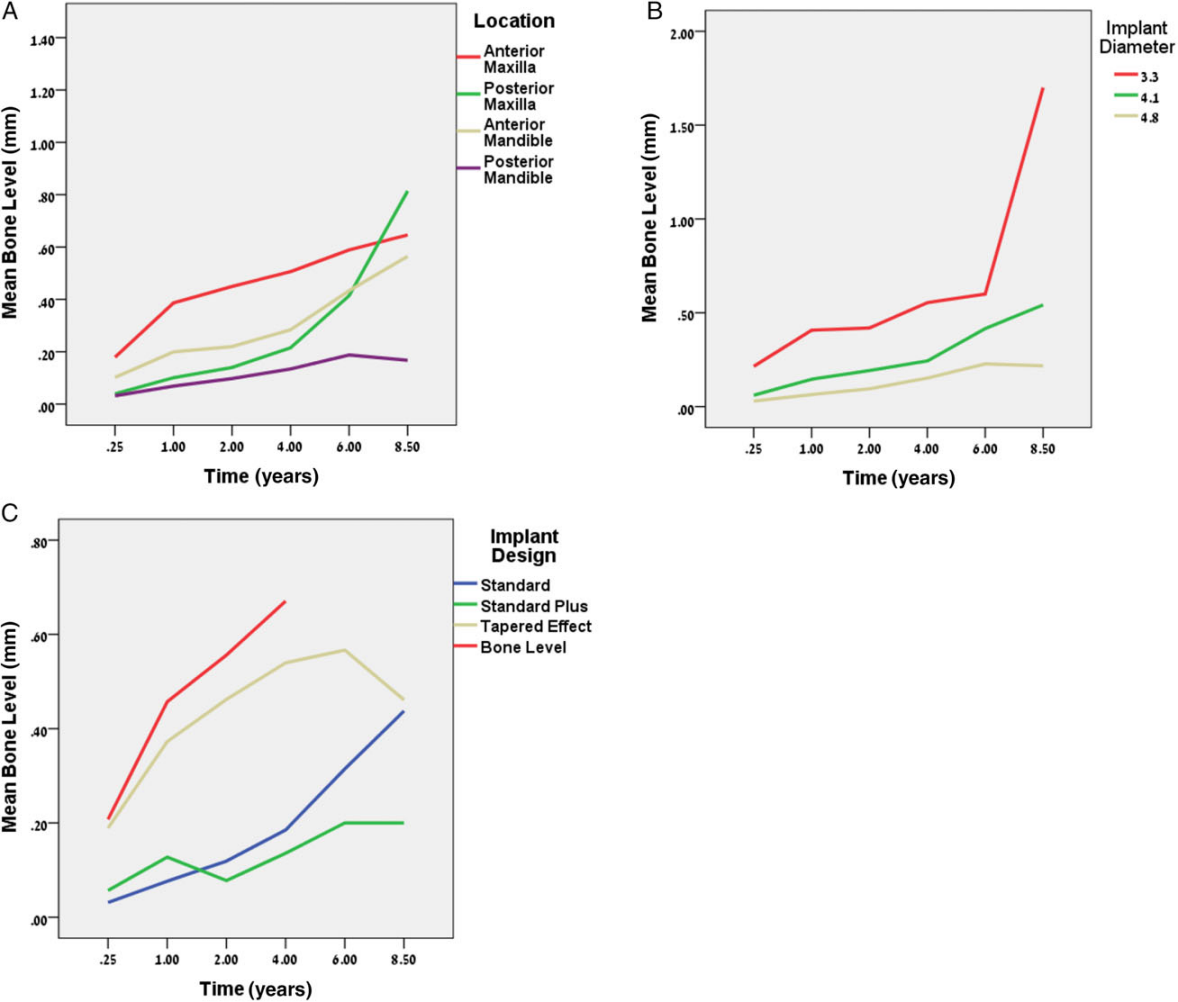
Mean crestal bone level (CBL) profiles for implant‐related factors **A**) location,^*^ **B**) diameter, and **C**) design. NB: y‐axis is not the same for different panels *Anterior maxilla as American Dental Association tooth # 6 to #11 and anterior mandible as tooth #21 to #28^49^

**Figure 3:**
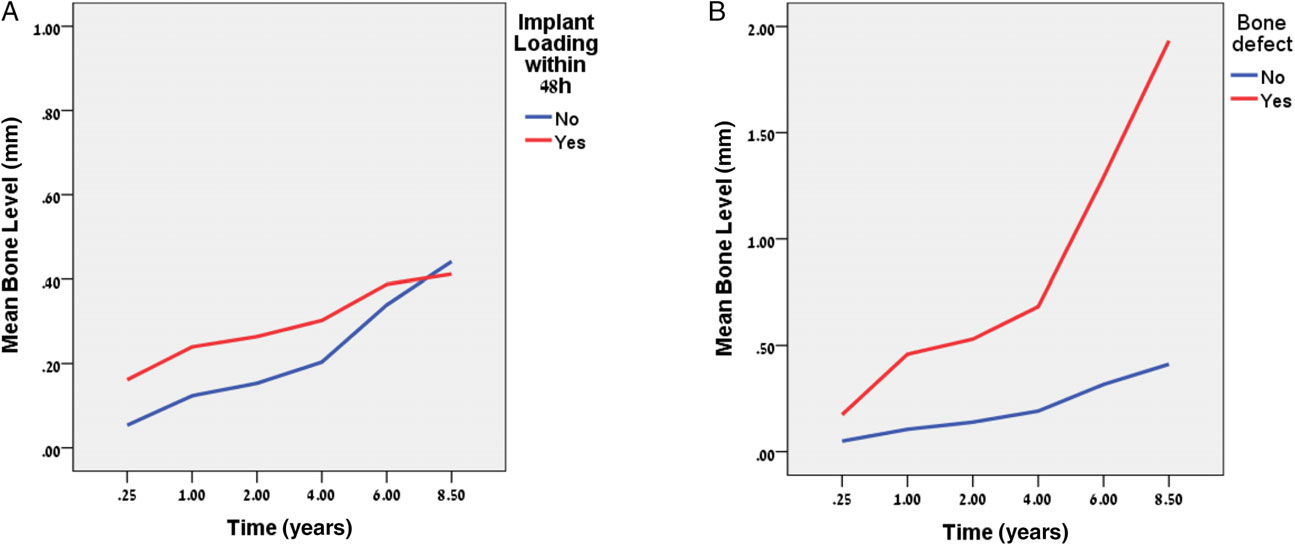
Mean crestal bone level (CBL) profiles for surgically related factors **A**) timing, and **B**) bone defect. NB: y‐axis is not the same for different panels

### 3.2 Patient‐related risk indicators

No significant effect on CBL was observed for diabetes (type 1 and type 2) nor history of periodontal disease.

#### 3.2.1 Autoimmune disease

There was no difference in initial CBL at the start (Figure 1A) but an almost significant main effect was observed overall (*P* value_ _= 0.04).

#### 3.2.2 Smokers

At the start there was no difference with regard to CBL between heavy smokers and non‐smokers (Figure 1B) but a significant main effect was found to exist (*P* value_ _<0.01) as well as a significant interaction with time. MBL among heavy smokers was more rapid after 4 years (Figure 1B).

#### 3.2.3 Bisphosphonates

There were 34 patients with 84 implants in this group with no implant failures (Figure 1C), although there were three sites with infections and also three other sites which required prolonged healing >6 months from stage 2 to pass torque test of 35 Ncm. An almost significant main effect (*P* value_ _= 0.045) with no difference at start existed among the bisphosphonate group.

### 3.3 Implant‐related risk indicators

The implant‐related factors all showed an effect as follows:

#### 3.3.1 Implant location

A significant difference in mean CBL existed as a function of implant location (Figure 2A) already at the start (*P* value_ _<0.01) with greater mean CBL for implants located anteriorly, compared with posterior mandible and posterior maxilla (see time = 0.25 in Figure 2A).

#### 3.3.2 Implant diameter

There was a significant difference in CBL as a function of implant diameter (Figure 2B) at the start, as well as a significant main effect and interaction with time.

#### 3.3.3 Implant design

Pairwise comparisons for CBL at the start between the four implant design groups revealed a similarity between standard and standard plus implants as well as between bone level and tapered effect implants (Figure 2C). However, a significant difference (*P* value_ _<0.01) was seen at the start between the two pairs with a greater mean loss observed for bone level and tapered effect implants.

### 3.4 Surgically related risk indicators

#### 3.4.1 Immediate loading

A significant difference for mean CBL at the start with a significant main effect and a non‐significant interaction with time was observed as a function of loading time (Figure 3A). Loading within 48 hours related to greater CBL but there was no difference between groups.

#### 3.4.2 Bone defect

A significant increase in MBL was associated with implants inserted into bone with a defect compared with native bone (Figure 3B). A significant difference was already present at the start, with a greater mean CBL for implants inserted into a bone defect site as well as a significant main effect and interaction effect (*P* value_ _<0.01).

### 3.5 The prevalence of mucositis and peri‐implantitis at implant and patient level

Tables 2 and 3 (see also supplementary Figs. 2 and 3 in online *Journal of Periodontology*) present the results at the implant level according to the strict and relaxed criteria. The number of healthy implants decreased over time. Using the strict criterion, the prevalence of peri‐implantitis increased from 0.4% at 2 to 3 years to 7.7% by 8 to 10 years (Table 2). The prevalence according to the relaxed criteria were lower (Table 3) showing only 5.9% at 8 to 10 years. At the patient level the prevalence of peri‐implantitis at 8 to 10 years was 11.7% and 7.8% for strict and relaxed, respectively.

**Table 2:** Implant level: Strict estimation of mucositis and peri‐implantitis prevalence

		Mucositis		
Bone Loss		No [IMI = 0]	Yes [IMI≥1]	Total
Bone loss ≥1 mm at 2 to 3 years	No Yes Total	1478[63.8%] 7 [0.3%] 1485	823 [35.5%] 10 [0.4%] 833	2301 17 2318
Bone loss ≥1 mm at 4 to 5 years	No Yes Total	708 [61%] 6 [0.5%] 714	424 [36.5%] 23 [2.0%] 447	1132 29 1161
Bone loss ≥1 mm at 6 to 7 years	No Yes Total	212 [54.9%] 7 [1.8%] 219	149 [38.6%] 18 [4.7%] 167	361 25 386
Bone loss ≥1 mm at 8 to 10 years	No Yes Total	92 [54.4%] 4 [2.4%] 96	60 [35.5%] 13 [7.7%] 73	152 17 169

**Table 3:** Implant level: Relaxed estimation of mucositis and peri‐implantitis prevalence

		Mucositis		
Bone loss		No (IMI = 0 or 1)	Yes (IMI ≥2)	Total
Bone loss ≥1 mm at 2 to 3 years	No Yes Total	2049 [88.4%] 7 [0.3%] 2056	252 [10.9%] 10 [0.4%] 262	2301 17 2318
Bone loss ≥1 mm at 4 to 5 years	No Yes Total	993 [85.5%] 8 [0.7%] 1001	139 [12.0%] 21 [1.8%] 160	1132 29 1161
Bone loss ≥1 mm at 6 to 7 years	No Yes Total	306 [79.3%] 11 [2.8%] 317	55 [14.2%] 14 [3.6%] 69	361 25 386
Bone loss ≥1 mm at 8 to 10 years	No Yes Total	137 [81.1%] 7 [4.1%] 144	15 [8.9%] 10 [5.9%] 25	152 17 169

## 4 DISCUSSION

An analysis of risk indicators for changes in crestal bone level, as a measure of bone loss (MBL), surrounding dental implants has been reported in this study. Furthermore, the impact of the choice of thresholds in determining prevalence of mucositis and peri‐implantitis has been considered. Importantly, this private practice report includes conventional patients and sites as well as at risk patients and at risk sites, therefore, unlike controlled studies with stringent selection criteria, it may better reflect the expected outcomes in daily clinical practice.^1^, ^24^

The following patient related risk indicators were found to be significant with regard to MBL: autoimmune disease, smoking and bisphosphonate use. Patients taking steroids for other chronic conditions were pooled, at the patient level, with patients having active autoimmune disease. The presence of steroid use or autoimmune disease had no impact on early bone scores but then showed increasing MBL over time (Figure 1A). The effect on MBL may result from the impaired bone healing owing to the osteopenic effect of steroids 25, 26 or the immune suppression in a manner similar to that seen on the rate of periodontal disease in patients with rheumatoid arthritis.^27^ Furthermore, this may lead to the occurrence of sporadic infection events, which may have an outlier effect on average MBL; however, conclusions drawn were limited by the small sample of patients in this category.

Regarding smoking, the results confirmed that heavy smoking can have a significant effect on MBL (Figure 1B). However, this result may be limited by the low prevalence of heavy smokers, at less than 2%, which is low by international standards, but is the expected rate for a high socioeconomic status (SES) cohort in Canada.^28^

As previously published in this cohort^21^ and in other studies,^29^ bisphosphonate therapy for osteoporosis, did not impact survival. However it did pose as a significant risk for MBL over time (Figure 1C). This is a unique finding in the literature and may reflect altered remodeling potential of bone, or it may also be the effect of a few outlier cases where sudden MBL was noted in some but not all bisphosphonate cases. Conclusions drawn are limited however as the duration or dose of bisphosphonate therapy was not recorded in this study.

Interestingly, diabetes (pooled type 1 and type 2) was not found to have a significant effect on MBL. However, the majority were type 2 and the average follow‐up was <4 years, therefore some diabetic cases may yet succumb to further MBL. More patients and a longer follow‐up time are needed to better assess this result. Similarly, a history of periodontal disease (pooled chronic and aggressive) was not found to have a significant effect on MBL in this study. However, the protocol for implant placement in periodontally involved patients was that the site of extraction had healed completely and thorough pre‐surgical root planing and regular recall was established. Conclusions are limited by an average of 4 years follow‐up, which may not be enough to reflect the risk and that oral hygiene was not directly evaluated as a risk indicator, it was only observed indirectly via the soft‐tissue IMI score.^8^

As for implant factors, including location, diameter and design, all were found to be significant with regard to marginal MBL. A difference in CBL at the start was observed in various locations with the most MBL found early on at the maxillary anterior sites. This may relate to the thin crestal bone remodeling at these sites or also to the use of bone graft for esthetic augmentation. The posterior mandible and posterior maxilla had equivalent crestal bone levels at the start but then the rate of MBL in the posterior maxilla was found to increase at a faster rate when compared with other locations (Figure 2A). This may be a result of crestal compression in lower density bone leading to MBL as described in finite element analysis.^30^, ^31^, ^32^ Indeed it was noted that about 6% of sites had MBL that could not be directly attributed to inflammation with sites exhibiting MBL despite negative bleeding scores overtime (see blue band in supplementary Figs. 2 and 3 in online *Journal of Periodontology*).

An inverse relationship between implant diameter and bone loss was observed, whereby an increase of 1 mm in diameter was associated with a decrease in CBL by about 0.11 mm (Figure 2B). Though narrow implant diameters led to more MBL early on, they did not lead to a higher rate of bone loss over time, as seen by the slope in Figure 2B. Narrow implants are typically placed in either narrow ridges or narrow proximal spaces of lower incisors and upper lateral incisors, which also typically have a reduced bucco‐lingual bone dimension. It is possible that the difference in MBL seen at narrow implants is an effect of remodeling of thin crestal bone. It is also possible that the steeper emergence profile at the neck of the RN platform on the narrow body parallel wall 3.3‐mm implant had potential crestal compression during implant seating.

Regarding implant design, it is important to consider the initial MBL expected for each implant design. The 1‐stage tissue level regular neck (RN) and wide neck (WN) implants, with 1.8‐mm and 2.8‐mm machined collars, are not affected by the microgap and typically have minimal MBL, i.e.: <0.5 mm.^13^, ^33^ However, the one‐stage tapered effect (TE) design was found to have increased initial MBL at base line (stage 2) (Figure 2C). For the TE design, the increased early MBL may be related to bone compression and a learning curve associated with placement of tapered implants.^12^ Two stage platform switch implants typically have bone loss of about 0.5 mm.^34^^‒^ 36 For the bone level platform shift design, the initial MBL of about 0.2 mm was better than expected for a platform shift design (Figure 2C). Of note is that, despite the initial increase in MBL for the bone level design, the subsequent rate of change is comparable to the RN and WN polished collar designs.

Of the surgically related risk indicators, immediate implant loading and presence of a bone defect with bone grafting were found to have an effect on MBL. Immediate loading, within 48 hours, is found to have a significant effect on early MBL, in this case 0.08 mm (Figure 3A). This small increase in radiographic MBL may be explained by load concentrated at the crestal bone caused by micro‐mobility of early loaded non‐integrated implants.^37^, ^38^ This may re‐mineralize as the implant becomes integrated and the micro‐mobility is reduced. Indeed, crestal bone seems to re‐establish over time as seen in Figure 3A, the slopes eventually intersect, which indicates that the early MBL is recovered and that the rate of MBL is not related to immediate loading. This is in keeping with other studies that found bone loss was not related to immediate loading despite having an effect on implant survival.^39^

Bone grafting of an osseous defect at the time of implant placement was also found to be a significant risk indicator for bone loss, as evidenced by the similarity of initial bone loss together with a significant interaction over time (different slopes) shown in Figure 3B with more MBL in grafted sites when compared with native bone. This is in support of systematic reviews of ridge augmentation that often show some loss of grafted bone volume and furthermore, it has been speculated that although the bone graft at time of placement may provide stable hard tissue, the basal bone may be the actual bone that is integrated to the implant.^40^^‒^ 42

### 4.1 Effect of threshold selection on reported prevalence of peri‐implantitis

The choice of appropriate threshold in assessing prevalence of peri‐implantitis is challenging, as evidenced by the range of reported estimates for peri‐implantitis from 1% to 47%,^43^, ^44^ with a 2012 EAO consensus reporting prevalence to be on the order of 10% of implants and 20% of patients.^5^ Prevalence is typically determined by cross‐tabulating bleeding‐on‐probing scores (BOP) with MBL, thus it is important to consider how each is determined.

BOP scores are typically recorded as either bleeding is present or absent and are used as an indicator for mucositis. Most studies, according to the EAO consensus of 2012,^5^, ^45^ used this simple binary BOP score plus suppuration, while others used a binary BOP with no reference to suppuration while only one study used the ordinal sulcus bleeding index (SBI)^46^. Dental implants, however, tend to bleed upon probing more frequently and at lower thresholds of probing force than teeth.^47^ Indeed in this current cohort, BOP was never below 35% at the implant level and it was ≥ 45% at the patient level over all time points.^8^ Therefore an ordinal scale may offer more specific information about the peri‐implant soft tissue and in this study a modification of the Sulcus Bleeding Index (SBI) which included suppuration, termed the Implant Mucosal Index (IMI), was used. The IMI has previously been shown to be a useful method to assess inflammation and, further, to relate each increase in IMI score with a doubling of MBL.^8^ In this study, prevalence of mucositis varied from nearly 50% of patients using a “strict” BOP threshold (IMI ≥1) (49.5%) compared with 18.2% if using the “relaxed” IMI threshold (IMI ≥2) (see supplementary Tables 3 and 4 in online *Journal of Periodontology*).

As the implants used in the current study were either platform shift or 1‐stage design <0.5 mm early MBL was expected to occur so a ≥1 mm MBL threshold was used as the MBL threshold. This is comparable to the threshold level of 1.2 mm loss beyond smooth‐rough interface with implants of similar design.^22^ Rodrigo et al. also evaluated MBL and recorded the highest score from mesial or distal sites, as was the method used in the current study.^15^

Taking both soft‐tissue and MBL thresholds into account, the prevalence of peri‐implantitis determined in this retrospective study, using a criteria of a ≥1 mm MBL and a “strict” BOP score, was found to be 4.7% after 6 to 7 years (Table 2), while the more “relaxed” soft‐tissue threshold (IMI≥2), excluding minor bleeding, resulted in a rate of 3.6% at 6 to 7 years (Table 3). A study of similar design also found a similar rate of peri‐implantitis and mucositis.^48^ In their study the prevalence of mucositis, as determined by probing depth >3 mm and BOP+ but no concomitant bone loss was 48% of implants. This is similar in that about half of the cases in the current study had some bleeding but not necessarily MBL. In the Roos‐Jansåker et al. study peri‐implantitis was defined as exposure of ≥3 threads (1.8‐mm MBL) with BOP or suppuration and they revealed a fairly comparable prevalence of peri‐implantitis at 7% for implant level.

One limitation in this study is that probing depths were not used, however these vary with soft tissue thickness, abutment and prosthetic design so are not easily compared between studies or between patients. The main limitation of this study is its retrospective nature with greater potential for missing data. Nevertheless, the high number of implants and the long follow‐up provides important insights into the clinical outcomes that one can expect in private practice. Another limitation of this study is the number of patients lost during follow up, which limits the ability to draw conclusions beyond 4 to 5 years. Furthermore, no intra‐examiner calibrations were done and all measurements were carried out by the clinician who placed the implants, thereby introducing a potential bias.

## 5 CONCLUSION

This open cohort, retrospective study evaluated risk indicators associated with marginal bone loss (MBL) through the analysis of 4,591 dental implants, of various designs, placed in private practice and followed‐up for 5 to 10 years. Significant risk indicators for bone loss were found to include autoimmune disease, heavy smoking, bisphosphonate therapy, implant location, diameter and design, and the presence of a bone defect at the site of placement. This study, using an ordinal scale for assessment of soft‐tissue conditions, reported that the prevalence of mucositis at the implant level, at 6 to 7 years, was higher at 38.6% versus 14.2% using strict versus relaxed criteria, respectively. The prevalence of peri‐implantitis was found to be 4.7% and 3.6%, using strict versus relaxed criteria, respectively. The results of this study highlight factors to consider when trying to prevent bone loss and further acknowledge the wide range of reported cases of peri‐implantitis and the need for universal standards.

## Supporting information

Supplementary Information for manuscript: Click here for additional data file.

Supplementary Table 1 Description of patient related risk indicators for bone loss

Supplementary Table 2 Summary of Implant Mucosal Index (IMI) as modified from sulcus bleeding index.

Supplementary Table 3 Patient level: Strict estimation of mucositis and peri‐implantitis prevalence.

Supplementary Table 4 Patient level: Relaxed estimation of mucositis and peri‐implantitis prevalence.

Supplementary Figure 1 For tissue level design implants (A), crestal bone level (CBL) is measured from the micro‐rough surface, where DIB is the distance from implant shoulder to the first bone to implant contact and NL is neck length (standard = 2.8 mm) or (standard plus = 1.8 mm). For the bone level design (B), CBL is measured from the implant neck. Marginal bone loss (MBL) is defined as a change in CBL from between subsequent time points, using stage 2, i.e.: 3 months post installation, as the baseline.

Supplementary Figure 2 The prevalence of mucositis and peri‐implantitis at implant level, [strict estimate].

Supplementary Figure 3 The prevalence of mucositis and peri‐implantitis at implant level, [relaxed estimate].

## Footnotes

1 Straumann Dental Implant System, Institute Straumann, Basel, Switzerland.

2 Straumann Dental Implant System.

3 Florida Probe, Gainesville, FL

4 IBM, Version 19.0, Armonk, NY

5 R Foundation for Statistical Computing, Vienna, Austria

## References

[1] Pjetursson BE , Asgeirsson AG , Zwahlen M , Sailer I . Improvements in implant dentistry over the last decade: comparison of survival and complication rates in older and newer publications. Int J Oral Maxillofac Implants. 2014;29(Suppl):308‐324. 24660206 10.11607/jomi.2014suppl.g5.2

[2] Albrektsson T , Donos N . Implant survival and complications. The third EAO consensus conference 2012. Clin Oral Implants Res. 2012;23:63‐65. 23062128 10.1111/j.1600-0501.2012.02557.x

[3] Atieh MA , Alsabeeha NHM , Faggion CM , Duncan WJ . The frequency of peri‐implant diseases: a systematic review and meta‐analysis. J Periodontol. 2013;84:1586‐1598. 23237585 10.1902/jop.2012.120592

[4] Abrahamsson I , Berglundh T . Effects of different implant surfaces and designs on marginal bone‐level alterations: a review. Clin Oral Implants Res. 2009;20(Suppl 4):207‐215. 19663966 10.1111/j.1600-0501.2009.01783.x

[5] Klinge B , Meyle J . Peri‐implant tissue destruction. The Third EAO Consensus Conference 2012. Clin Oral Implants Res. 2012;23:108‐110. 23062134 10.1111/j.1600-0501.2012.02555.x

[6] Lindhe J , Meyle J . Peri‐implant diseases: consensus report of the sixth European workshop on periodontology. J Clin Periodontol. 2008;35:282‐285. 18724855 10.1111/j.1600-051X.2008.01283.x

[7] Mombelli A , Oosten MAC , Schürch E , Lang NP . The microbiota associated with successful or failing osseointegrated titanium implants. Oral Microbiol Immunol. 1987;2:145‐151. 3507627 10.1111/j.1399-302x.1987.tb00298.x

[8] French D , Cochran D , Ofec R . Retrospective cohort study of 4591 Straumann implants placed in 2060 patients in private practice with up to 10 year follow‐up: the relationship between crestal bone level and soft tissue condition. Int J oral Maxillofac Implant. 2016;31:e168‐e178. 10.11607/jomi.4932 27861661

[9] Heitz‐Mayfield LJA . Peri‐implant diseases: diagnosis and risk indicators. Journal of Clinical Periodontology. 2008;35:292‐304. 18724857 10.1111/j.1600-051X.2008.01275.x

[10] Quirynen M , De Soete M , van Steenberghe D . Infectious risks for oral implants: a review of the literature. Clin Oral Implants Res. 2002;13:1‐19. 12005139 10.1034/j.1600-0501.2002.130101.x

[11] Albouy J‐P , Abrahamsson I , Persson LG , Berglundh T . Spontaneous progression of peri‐implantitis at different types of implants. An experimental study in dogs. I: clinical and radiographic observations. Clin Oral Implants Res. 2008;19:997‐1002. 18828815 10.1111/j.1600-0501.2008.01589.x

[12] Ho DSW , Yeung SC , Zee KY , Curtis B , Hell P , Tumuluri V . Clinical and radiographic evaluation of NobelActive(TM) dental implants. Clin Oral Implants Res. 2013;24:297‐304. 22092589 10.1111/j.1600-0501.2011.02313.x

[13] Laurell L , Lundgren D . Marginal bone level changes at dental implants after 5 years in function: a meta‐analysis. Clin Implant Dent Relat Res. 2011;13:19‐28. 19681932 10.1111/j.1708-8208.2009.00182.x

[14] Poli PP , Beretta M , Grossi GB , Maiorana C . Risk indicators related to peri‐implant disease: an observational retrospective cohort study. J Periodontal Implant Sci. 2016;46:266‐276. 27588216 10.5051/jpis.2016.46.4.266 PMC5005814

[15] Rodrigo D , Martin C , Sanz M . Biological complications and peri‐implant clinical and radiographic changes at immediately placed dental implants. A prospective 5‐year cohort study. Clin Oral Implants Res. 2012;23:1224‐1231. 22092726 10.1111/j.1600-0501.2011.02294.x

[16] Stavropoulos A , Cochran D , Obrecht M , Pippenger BE , Dard M . Effect of osteotomy preparation on osseointegration of immediately loaded, tapered dental implants. Adv Dent Res. 2016;28:34‐41. 26927486 10.1177/0022034515624446

[17] Tarnow DP , Cho SC , Wallace SS . The effect of inter‐implant distance on the height of inter‐implant bone crest. J Periodontol. 2000;71:546‐549. 10807116 10.1902/jop.2000.71.4.546

[18] Elian N , Bloom M , Dard M , Cho SC , Trushkowsky RD , Tarnow D . Radiological and micro‐computed tomography analysis of the bone at dental implants inserted 2, 3 and 4 mm apart in a minipig model with platform switching incorporated. Clin Oral Implants Res. 2014;25:e22‐e29. 23020736 10.1111/clr.12037

[19] Linkevicius T , Apse P , Grybauskas S , Puisys A . Influence of thin mucosal tissues on crestal bone stability around implants with platform switching: a 1‐year pilot study. J Oral Maxillofac Surg. 2010;68:2272‐2277. 20605308 10.1016/j.joms.2009.08.018

[20] Serino G , Turri A , Lang NP . Probing at implants with peri‐implantitis and its relation to clinical peri‐implant bone loss. Clin Oral Implants Res. 2013;24:91‐95. 22462625 10.1111/j.1600-0501.2012.02470.x

[21] French D , Larjava H , Ofec R . Retrospective cohort study of 4591 Straumann implants in private practice setting, with up to 10‐year follow‐up. part 1: multivariate survival analysis. Clin Oral Implants Res. 2015;26:1345‐1354. 25134415 10.1111/clr.12463

[22] Buser D , Janner SF , Wittneben JG , Brägger U , Ramseier CA , Salvi GE . 10‐year survival and success rates of 511 titanium implants with a sandblasted and acid‐etched surface: a retrospective study in 303 partially edentulous patients. Clin Implant Dent Relat Res. 2012;14:839‐851. 22897683 10.1111/j.1708-8208.2012.00456.x

[23] Ainamo J , Bay I . Problems and proposals for recording gingivitis and plaque. Int Dent J. 1975;25:229‐235. 1058834

[24] De Bruyn H , Vandeweghe S , Ruyffelaert C , Cosyn J , Sennerby L . Radiographic evaluation of modern oral implants with emphasis on crestal bone level and relevance to peri‐implant health. Periodontol 2000. 2013;62:256‐270. 23574471 10.1111/prd.12004

[25] Parrillo JE , Fauci AS . Mechanisms of glucocorticoid action on immune processes. Annu Rev Pharmacol Toxicol. 1979;19:179‐201. 222199 10.1146/annurev.pa.19.040179.001143

[26] Waters RV , Gamradt SC , Asnis P , et al. Systemic corticosteroids inhibit bone healing in a rabbit ulnar osteotomy model. Acta Orthop Scand. 2000;71:316‐321. 10919307 10.1080/000164700317411951

[27] Pischon N , Pischon T , Kröger J , et al. Association among rheumatoid arthritis, oral hygiene, and periodontitis. J Periodontol. 2008;79:979‐986. 18533773 10.1902/jop.2008.070501

[28] Reid JL , Hammond D , Driezen P . Socio‐economic status and smoking in Canada, 1999‒2006: has there been any progress on disparities in tobacco use. Can J Public Health. 2010;101:73‐78. 20364543 10.1007/BF03405567 PMC6973977

[29] Madrid C , Sanz M . What impact do systemically administrated bisphosphonates have on oral implant therapy? A systematic review. Clin Oral Implants Res. 2009;20(Suppl 4):87‐95. 10.1111/j.1600-0501.2009.01772.x 19663954

[30] Huang H‐L , Huang JS , Ko CC , Hsu JT , Chang CH , Chen MY . Effects of splinted prosthesis supported a wide implant or two implants: a three‐dimensional finite element analysis. Clin Oral Implants Res. 2005;16:466‐472. 16117772 10.1111/j.1600-0501.2005.01124.x

[31] Sevimay M , Turhan F , Kiliçarslan MA , Eskitascioglu G . Three‐dimensional finite element analysis of the effect of different bone quality on stress distribution in an implant‐supported crown. J Prosthet Dent. 2005;93:227‐234. 15775923 10.1016/j.prosdent.2004.12.019

[32] Yilmaz B , Seidt JD , McGlumphy EA , Clelland NL . Comparison of strains for splinted and nonsplinted screw‐retained prostheses on short implants. Int J Oral Maxillofac Implants. 2011;26:1176‐1182. 22167421

[33] Hänggi MP , Hänggi DC , Schoolfield JD , Meyer J , Cochran DL , Hermann JS . Crestal bone changes around titanium implants. Part I: a retrospective radiographic evaluation in humans comparing two non‐submerged implant designs with different machined collar lengths. J Periodontol. 2005;76:791‐802. 15898941 10.1902/jop.2005.76.5.791

[34] Annibali S , Bignozzi I , Cristalli MP , Graziani F , La Monaca G , Polimeni A . Peri‐implant marginal bone level: a systematic review and meta‐analysis of studies comparing platform switching versus conventionally restored implants. Journal of Clinical Periodontology. 2012;39:1097‐1113. 22931292 10.1111/j.1600-051X.2012.01930.x

[35] Atieh M , Ibrahim HM , Atieh AH . Platform switching for marginal bone preservation around dental implants: a systematic review and meta‐analysis. J Periodontol. 2010;81:1350‐1366. 20575657 10.1902/jop.2010.100232

[36] Strietzel FP , Neumann K , Hertel M . Impact of platform switching on marginal peri‐implant bone‐level changes. A systematic review and meta‐analysis. Clinical Oral Implants Research. 2015;26:342‐358. 24438506 10.1111/clr.12339 PMC4340042

[37] Frost HMA . 2003 update of bone physiology and Wolff's Law for clinicians. Angle Orthod. 2004;74:3‐15. 15038485 10.1043/0003-3219(2004)074<0003:AUOBPA>2.0.CO;2

[38] Frost HM . Wolff's law and bone's structural adaptations to mechanical usage: an overview for clinicians. Angle Orthod. 1994;64:175‐188. 8060014 10.1043/0003-3219(1994)064<0175:WLABSA>2.0.CO;2

[39] Testori T , Zuffetti F , Capelli M , Galli F , Weinstein RL , Del Fabbro M . Immediate versus conventional loading of post‐extraction implants in the edentulous jaws. Clin Implant Dent Relat Res. 2014;16:926‐935. 23506353 10.1111/cid.12055

[40] Chiapasco M , Colletti G , Coggiola A , Di Martino G , Anello T , Romeo E . Clinical outcome of the use of fresh frozen allogeneic bone grafts for the reconstruction of severely resorbed alveolar ridges: preliminary results of a prospective study. Int J Oral Maxillofac Implants. 2015;30:450‐460. 25830406 10.11607/jomi.3763

[41] Chiapasco M , Zaniboni M . Clinical outcomes of GBR procedures to correct peri‐implant dehiscences and fenestrations: a systematic review. Clin Oral Implants Res. 2009;20:113‐123. 19663958 10.1111/j.1600-0501.2009.01781.x

[42] Aghaloo TL , Moy PK . Which hard tissue augmentation techniques are the most successful in furnishing bony support for implant placement. Int J Oral Maxillofac Implants. 2007;22(Suppl):49‐70. 18437791

[43] Fischer K , Stenberg T . Prospective 10‐year cohort study based on a randomized controlled trial (RCT) on prostheses. Part 1 : sandblasted and acid‐etched implants and mucosal tissue. Clin Implant Dent Relat Res. 2012;14:1‐8. 22008715 10.1111/j.1708-8208.2011.00389.x

[44] Koldsland OC , Scheie AA , Aass AM . Prevalence of peri‐implantitis related to severity of the disease with different degrees of bone loss. J Periodontol. 2010;81:231‐238. 20151801 10.1902/jop.2009.090269

[45] Mombelli A , Müller N , Cionca N . The epidemiology of peri‐implantitis. Clin Oral Implants Res. 2012;23:67‐76. 10.1111/j.1600-0501.2012.02541.x 23062130

[46] Corbella S , Del Fabbro M , Taschieri S , De Siena F , Francetti L . Clinical evaluation of an implant maintenance protocol for the prevention of peri‐implant diseases in patients treated with immediately loaded full‐arch rehabilitations. Int J Dent Hyg. 2011;9:216‐222. 21356024 10.1111/j.1601-5037.2010.00489.x

[47] Gerber JA , Tan WC , Balmer TE , Salvi GE , Lang NP . Bleeding on probing and pocket probing depth in relation to probing pressure and mucosal health around oral implants. Clin Oral Implants Res. 2009;20:75‐78. 19126110 10.1111/j.1600-0501.2008.01601.x

[48] Roos‐Jansåker A‐M , Lindahl C , Renvert H , Renvert S . Nine‐ to fourteen‐year follow‐up of implant treatment. Part II: presence of peri‐implant lesions. J Clin Periodontol. 2006;33:290‐295. 16553638 10.1111/j.1600-051X.2006.00906.x

[49] Buser D , Mericske‐Stern R , Bernard JP , et al. Long‐term evaluation of non‐submerged ITI implants. Part 1: 8‐year life table analysis of a prospective multi‐center study with 2359 implants. Clin Oral Implants Res. 1997;8:161‐172. 9586460 10.1034/j.1600-0501.1997.080302.x

