# Supplementary material for: Retrospective cohort study of 4,591 dental implants: Analysis of risk indicators for bone loss and prevalence of peri‐implant mucositis and peri‐implantitis
Source: J Periodontol. 2019 Feb 6;90(7):691–700. doi: 10.1002/JPER.18-0236 (PMC6849729; doi:10.1002/JPER.18-0236)
Supplement: Supplementary file 1 — Supplementary Information for manuscript: [file JPER-90-691-s001.docx]

**Supplemental Information for manuscript:**

**Title:** Retrospective Cohort Study of 4591 Straumann Implants: Analysis of risk indicators for bone loss and prevalence of peri-implant mucositis and peri-implantitis

**Authors:**

David French*, DDS; H Michelle Grandin†, PhD: Ronen Ofec‡, DMD;

**Materials and Methods, Supplement:**

Further details of the study cohort including graphic presentation are in a previous publication.^1^ The inclusion criterion was the presentation of edentulous or partially edentulous sites and the only exclusion criterion was the use of ASA class 3 or higher.^2^ All potential implant locations were used, and the location of implants was determined based on individual patient’s requirements; no set location or group of locations were planned or declined. Patient education and consent to implant surgery was obtained. The study was part of an ongoing long-term evaluation of dental implants associated with a University of British Columbia retrospective clinical study on dental implants. The study was approved by the Clinical Research Ethics Board at the University of British Columbia (Vancouver, Canada # H13-01664 titled UBC Implants) and was conducted in accordance with the Helsinki Declaration of 1975, as revised in 2000. Data analysis was designed to preserve the anonymity of the patients. The study follows the STROBE guidelines for cohort studies.^3^

The cohort included 922 (44.8%) males and 1138 (55.2%) females with a mean age at surgery of 50.58 ± 12.96 years and a range of 15 to 85 years. Implants, irrespective of type and/or dimension, were distributed as follows: n=625 (13.7%) in the anterior maxilla, n=1717 (37.4%) in the posterior maxilla, n=291 (6.3%) in the anterior mandible and n=1958 (42.6%) in the posterior mandible. In the case of immediate implant placement in extraction sockets, the rough interface of the implant was placed at least 1mm sub-crestally in a fully intact socket and if the bone to implant gap was less than 1.5mm, the gap was not grafted. In the case of atrophic ridges, bone grafting was done simultaneously at the time of implant placement with a membrane and particulate bone as previously described.^1^ A description of patient related risk indicators is seen in Supplemental Table 1.

The implant mucosal index was applied as per Supplemental Table 2. Of note is IMI 1 was strictly minimal bleeding at a single point, however if there were multiple sites of bleeding, or if moderate bleeding or greater, it would be described as class 2 or higher.

**Statistics Methodology:**

The model used in this study accounts for the unique correlation structure within units of analysis as a reflection of the study design; measurements were repeated over time within implants that were nested within a patient. The following random components were included in the model: random patient specific effects associated with both the intercept and slope (i.e., the effect of time) for each patient, and random effects associated with the intercept for each implant nested within a patient. Based on the finding that variability of bone loss increases over time, a diagonal covariance structure for the residuals associated with repeated measurements on the same implant was included. This structure allowed for heterogeneous residual variances at each time point. The fixed effects in the model were included and univariate analysis was applied to identify factors associated with changes in CBL over time. Predictors with PVALUE<0.05, based on univariate analysis, were entered into a "loaded model" that was adjusted for the clustering structure of the data set. Non-significant variables were removed, one by one, in order to obtain a final parsimonious multivariate model.

Since bone level as the main response variable has a non-normal distribution, with many zeros and a right-skewed tail, a violation of model assumptions existed and could impair the validity of the results. To overcome this problem, the model was compared against a GEE logistic model (not shown in this manuscript) with crestal bone level as a binary outcome (cut off at 0 mm) and the same fixed effects. The results of both models were similar.

Two alternative calculations are presented: the first was the strict estimation, classifying IMI≥1 as mucositis and the second, the more relaxed estimation, classifying IMI≥2 as mucositis. In order to perform the same analysis at the patient level, new variables were defined at the patient level as follows: 1) The most severe IMI diagnosis within implants of a patient, 2) A binary variable to indicate if at least one implant within the patient was classified as having mucositis (according to strict and relaxed criteria) and 3) A binary variable to indicate if at least one implant within the patient was classified as presenting peri-implantitis (according to strict and relaxed criteria).

**Results, supplemental:**

See: Supplemental Figure 2 and 3 and Supplemental Tables 3 & 4.

**Table and Figure Captions, Supplemental:**

**Supplemental Table 1**. Description of patient related risk indicators for bone loss.

**Supplemental Table 2.** Summary of Implant Mucosal Index (IMI) as modified from sulcus bleeding index.

**Supplemental Table 3:** Patient level: Strict estimation of mucositis and peri-implantitis prevalence.

**Supplemental Table 4**. Patient level: Relaxed estimation of mucositis and peri-implantitis prevalence.

**Supplemental Figure 1**. For tissue level design implants (A), crestal bone level (CBL) is measured from the micro-rough surface, where DIB is the distance from implant shoulder to the first bone to implant contact and NL is neck length (standard =2.8mm) or (standard plus =1.8mm). For the bone level design (B), CBL is measured from the implant neck. Marginal bone loss (MBL) is defined as a change in CBL from between subsequent time points, using stage 2, i.e.: 3 months post installation, as the baseline.

**Supplemental Figure 2.** The prevalence of mucositis and peri-implantitis at implant level, [strict estimate].

**Supplemental Figure 3.** The prevalence of mucositis and peri-implantitis at implant level,

[relaxed estimate].

**References, Supplemental:**

1. French D, Larjava H, Ofec R. Retrospective cohort study of 4591 Straumann implants in private practice setting, with up to 10-year follow-up. Part 1: multivariate survival analysis. Clin Oral Implants Res. 2015 Nov;26(11):1345–54.

2. Owens WD, Felts JA, Spitznagel ELJ. ASA physical status classifications: a study of consistency of ratings. Anesthesiology. 1978 Oct;49(4):239–43.

3. von Elm E, Altman DG, Egger M, Pocock SJ, Gøtzsche PC, Vandenbroucke JP. The Strengthening the Reporting of Observational Studies in Epidemiology (STROBE) statement: guidelines for reporting observational studies. J Clin Epidemiol. 2008;61(4):344–9.

4. Reid JL, Hammond D, Driezen P. Socio-economic status and smoking in Canada, 1999-2006: has there been any progress on disparities in tobacco use? Can J Public Health. 2010;101(1):73–8.

5. Marx RE, Cillo JE, Ulloa JJ. Oral Bisphosphonate-Induced Osteonecrosis: Risk Factors, Prediction of Risk Using Serum CTX Testing, Prevention, and Treatment. J Oral Maxillofac Surg. 2007 Dec;65(12):2397–410.
