# Supplementary material for: Retrospective cohort study of 4,591 dental implants: Analysis of risk indicators for bone loss and prevalence of peri‐implant mucositis and peri‐implantitis
Source: J Periodontol. 2019 Feb 6;90(7):691–700. doi: 10.1002/JPER.18-0236 (PMC6849729; doi:10.1002/JPER.18-0236)
Supplement: Supplementary file 2 — Supplementary Table 1 Description of patient related risk indicators for bone loss [file JPER-90-691-s005.docx]

**Supplemental Table 1**. Description of patient related risk indicators for bone loss.

| Condition | Description | Number | Comment |
| --- | --- | --- | --- |
| Disease:  Autoimmune | Any autoimmune condition reported by patient and/ or long term systemic steroid medication. | 28 implants  14 patients | Examples included but not limited to Rheumatoid arthritis, Scleroderma, Dermatomyositis, Autoimmune Hepatitis,  Lupus. |
| Disease: Diabetes type 1 and 2. | Diabetic status as determined by self reporting and/or reporting of diabetes medication. | 76 implants (n=4 for type 1, n=72 for Type 2)  27 patients (n=4 for type 1, n=23 for type 2). | Uncontrolled diabetics were not treated. |
| Disease:  History of Periodontitis, | Combined Advanced  adult periodontitis & Generalized aggressive periodontitis with  ≥6mm in 4 quadrants | 189 implants  65 patients | Periodontal disease history subjects received periodontal treatment prior to implant therapy and remained on periodontal recall |
| Heavy Smoking | ≥15 cigarettes per day  self-reported  at any time during placement or follow up. | 84 implants  29 patients | Light smoking not recorded, pooled with non-smokers. Heavy smoking prevalence in this study population is within expected range for high SES group in Canada ** |
| Bisphosphonate | Bisphosphonate use was only related to osteoporosis.  Dose and durations not recorded however 33/34 were oral medication. 1 patient on IV Zometa. | 84 implants  34 patients | Subjects treated after 2007 were given a C-terminal telopeptide blood test (CTX)# and 3 month drug holiday if CTX < 150 pg/ml. |

** smoking rates in Canada^4^, # CTX as defined by Marx et al.^5^

Digital radiographs were taken using a proprietary parallel film holder and software calibrated to sensor dimensions (Dexis ,Hatfield, PA USA), standardized radiographic stents were not used. CBL was defined as DIB minus the neck length (NL) (that is the machined surface) of an implant (Supplemental Figure 1).
