# Supplementary material for: Retrospective cohort study of 4,591 dental implants: Analysis of risk indicators for bone loss and prevalence of peri‐implant mucositis and peri‐implantitis
Source: J Periodontol. 2019 Feb 6;90(7):691–700. doi: 10.1002/JPER.18-0236 (PMC6849729; doi:10.1002/JPER.18-0236)
Supplement: Supplementary file 3 — Supplementary Table 2 Summary of Implant Mucosal Index (IMI) as modified from sulcus bleeding index. [file JPER-90-691-s006.docx]

**Supplemental Table 2.** Summary of Implant Mucosal Index (IMI) as modified from sulcus bleeding index.

| IMI | Bleeding on probing* |
| --- | --- |
| 0 | No bleeding |
| 1 | Minimal, single point bleeding |
| 2 | Moderate, multi point bleeding |
| 3 | Profuse, multi point bleeding |
| 4 | Suppuration |
| * probing 6 sites, probe 17 gram | |
