# Supplementary material for: Retrospective cohort study of 4,591 dental implants: Analysis of risk indicators for bone loss and prevalence of peri‐implant mucositis and peri‐implantitis
Source: J Periodontol. 2019 Feb 6;90(7):691–700. doi: 10.1002/JPER.18-0236 (PMC6849729; doi:10.1002/JPER.18-0236)
Supplement: Supplementary file 5 — Supplementary Table 4 Patient level: Relaxed estimation of mucositis and peri‐implantitis prevalence. [file JPER-90-691-s008.docx]

**Supplemental Table 4**. Patient level: Relaxed estimation of mucositis and peri-implantitis prevalence.

| Follow-up | Mucositis* | Peri-implantitis* | N |
| --- | --- | --- | --- |
| 2-3 years | 14.9% | 0.8% | 1036 |
| 4-5 years | 17.7% | 2.7% | 525 |
| 6-7 Years | 21.0% | 4.8% | 186 |
| >= 8 Years | 18.2% | 7.8% | 77 |
| * Not mutually exclusive | | | |
