# Supplementary figures and images for: Retrospective cohort study of 4,591 dental implants: Analysis of risk indicators for bone loss and prevalence of peri‐implant mucositis and peri‐implantitis
Source: J Periodontol. 2019 Feb 6;90(7):691–700. doi: 10.1002/JPER.18-0236 (PMC6849729; doi:10.1002/JPER.18-0236)

A) B)


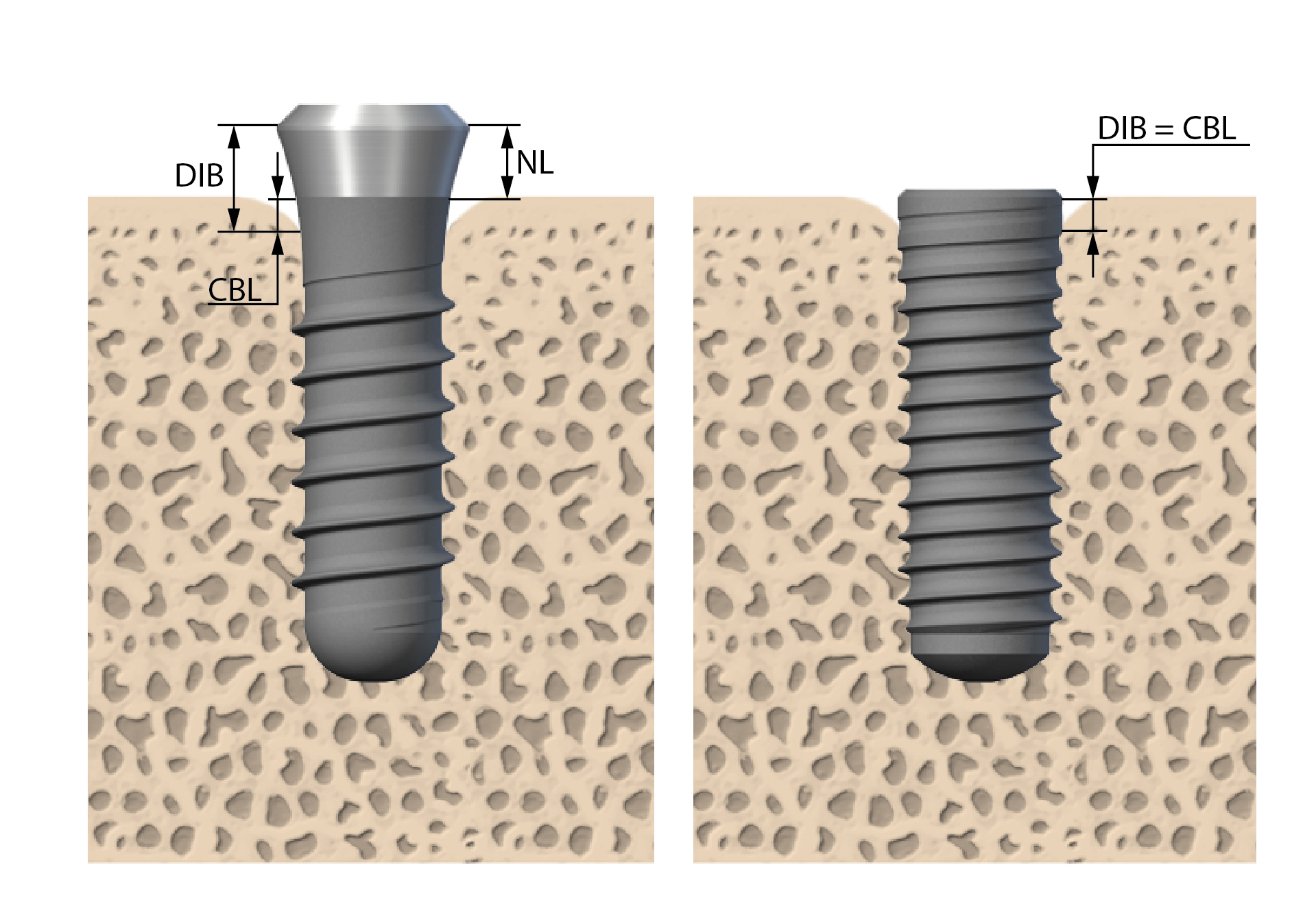

Supplement: Supplementary file 6 — Supplementary Figure 1 For tissue level design implants (A), crestal bone level (CBL) is measured from the micro‐rough surface, where DIB is the distance from implant shoulder to the first bone to implant contact and NL is neck length (standard = 2.8 mm) or (standard plus = 1.8 mm). For the bone level design (B), CBL is measured from the implant neck. Marginal bone loss (MBL) is defined as a change in CBL from between subsequent time points, using stage 2, i.e.: 3 months post installation, as the baseline. [file JPER-90-691-s002.docx]
