# Supplementary material for: Retrospective cohort study of 4,591 dental implants: Analysis of risk indicators for bone loss and prevalence of peri‐implant mucositis and peri‐implantitis
Source: J Periodontol. 2019 Feb 6;90(7):691–700. doi: 10.1002/JPER.18-0236 (PMC6849729; doi:10.1002/JPER.18-0236)
Supplement: Supplementary file 7 [file JPER-90-691-s003.docx]

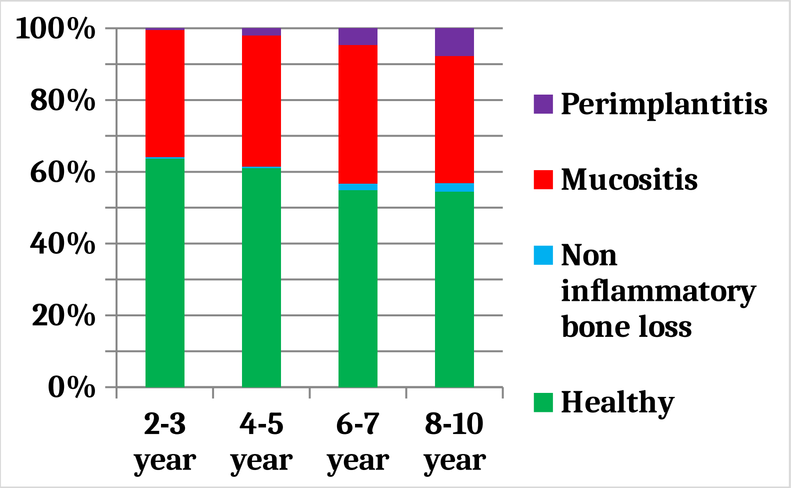
100%
80%
60%
40%
20%
0%
2-3 year
4-5 year
6-7 year
8-10 year
Perimplantitis
Mucositis
Non inflammatory bone loss
Healthy
